# Influence of Internal Innovative Architecture on the Mechanical Properties of 3D Polymer Printed Parts

**DOI:** 10.3390/polym12051129

**Published:** 2020-05-14

**Authors:** Mihai Alin Pop, Cătălin Croitoru, Tibor Bedo, Virgil Geamăn, Irinel Radomir, Sebastian Marian Zaharia, Lucia Antoaneta Chicoș

**Affiliations:** 1 Materials Science Department, Transilvania University of Brasov, 29 Eroilor Ave., 500036 Brasov, Romania; bedo.tibor@unitbv.ro (T.B.); geaman.v@unitbv.ro (V.G.); 2 Materials Engineering and Welding Department, Transilvania University of Brasov, 29 Eroilor Ave., 500036 Brasov, Romania; 3 Mathematics and Informatics Department, Transilvania University of Brasov, 29 Eroilor Ave., 500036 Brasov, Romania; i.radomir@unitbv.ro; 4 Manufacturing Engineering Department, Faculty of Technological Engineering and Industrial Management, Transilvania University of Brasov, 29 Eroilor Ave., 500036 Brasov, Romania; zaharia_sebastian@unitbv.ro (S.M.Z.); l.chicos@unitbv.ro (L.A.C.)

**Keywords:** additive manufacturing, 3D printing, Anderson–Darling statistical test, mechanical properties, polymers, composite materials

## Abstract

The utilization of polymer-based materials is quickly expanding. The enterprises of today are progressively seeking techniques to supplant metal parts with polymer-based materials as a result of their light weight, simple support and modest costs. The ceaselessly developing requirement for composite materials with new or enhanced properties brings about the preparation of different polymer mixes with various arrangements, morphologies and properties. Fused filament fabrication processes such as 3D-printing are nowadays shaping the actual pathway to a full pallet of materials, from art–craft to biomaterials. In this study, the structural and mechanical behavior of three types of commercially available filaments comprised of synthetic poly(acrylonitrile-co-butadiene-co-styrene) (ABS), poly(lactic acid) (PLA) and poly(lactic acid)/polyhydroxyalkanoate reinforced with bamboo wood flour composite (PLA/PHA BambooFill) were assessed through mechanical testing and optical microscopy, aiming to understand how the modifications that occur in the printed models with internal architecture are influencing the mechanical properties of the 3D-printed material. It has been determined that the material printed from PLA presents the highest compression strength, three-point bending and shock resistance, while the ABS shows the best tensile strength performance. A probability plot was used to verify the normality hypothesis of data for the tensile strength, in conjunction with the Anderson–Darling statistic test. The results of the statistic indicated that the data were normally distributed and that there is a marked influence of the internal architecture of the 3D-printed models on the mechanical properties of the printed material.

## 1. Introduction

Additive manufacturing represents a group of technological processes which all have the building of three-dimensional (3D) objects by adding materials layer by layer from a computer-aided design (CAD) model of the object in common [1,2]. The feedstock materials can be in different states, such as powder, liquid, or sheet form. Among these processes, powder bed fusion (including selective laser sintering and laser cladding) has been well implemented commercially since the 1980s for building workpieces with a diverse structural pallet. Initially limited to metallic powders, or metal–ceramic composite formulations, additive manufacturing has rapidly shifted to other types of materials, such as ceramics, polymers and polymer-matrix composites [2,3,4]. New variants of additive manufacturing are emerging and beginning to be commercially implemented, such as microstereolithography and fused filament fabrication (FFF), the latter of which is also known as 3D printing. The 3D printing process allows for the obtaining of complex products with low environmental impact, material loss and production costs compared to conventional methods (drilling, milling, extrusion, injection molding, etc.) [5,6]. It also has one of the most extensive application palettes among the additive manufacturing processes, ranging from typical consumer goods to specialty applications such as microelectronic and microfluidic devices, electrodes, interfaces, biomaterials, and so forth [6,7,8].

One serious drawback of the 3D printing method, mainly when applied to polymer and composite materials, is represented by the relatively low mechanical properties of the build, comparing to those of the materials obtained through conventional forming processes (e.g., extrusion, injection and thermoforming) [5,9]. Numerous research has been conducted to improve the mechanical properties of extruded 3D-printed materials, which could be grouped in two main directions: developing new type of filaments and optimizing the printing geometry of the material. Regarding the first approach, many commercial filaments are already available or under development, which make use of several types of polymer blends (PLA/PHA [10,11,12,13], PC/ABS [14], PU/PLA [14], etc.) and composites (polymer–carbon nanotubes [11,15], polymer–ceramic [15,16], polymer-metal [16], polymer-wood [17], etc.) formulations. Furthermore, to improve the stability of the polymer, melt during extrusion, the introduction of additives into the filament receipt, like coupling agents, plasticizers, stabilizers, etc., could tune the mechanical response to several degradative factors of the final 3D-printed polymer [5,18,19].

To date, the mechanical behavior of 3D-printed structures originating from several types of filaments [20], the influence of build defects on the properties of the material or optimization of the layer height, printing duration and material consumption have already been extensively reported, mainly based on PLA as a filament material [21,22,23]. The structural, thermodynamic and mechanical properties of the polymeric 3D-printed structures vary depending upon their chemical or biological composition, but they typically emerge as high-versatility materials with a range of interdisciplinary applications.

However, relatively little research has been conducted studying the relationship between the internal geometry (type of infill patterns and cross-section) of the CAD model and its 3D-printed counterpart mechanical properties, or between the model’s topology and material consumption [22,23,24,25]. There are a variety of infill patterns available: hexagonal, concentric, rectilinear, Hilbert curve and Archimedean chords, which are used to print “solid” builds at various infill percentages. Obviously, a solid-printed material will present the best mechanical properties, but as well as the extent of high material consumption and build duration [25]. One of the challenges of 3D printing is represented by optimizing the topology of the material to achieve the best weight-specific mechanical properties, at the lowest extent of consumed material [17,26]. 

The novelty of this work resides in developing and characterizing two internal build architectures for 3D-printed materials, namely a square model structure with 1-mm wall thickness and a tubular architecture with a diameter of 1.6 mm and with 2 mm distance between the axes x, y and z, aiming towards high-tensile, 3-point bending, compression and impact resistance properties at the lowest possible densities and amounts of materials consumed. The internal structure of the 3D-printed materials and their porosity are studied by optical microscopy. The energy absorbed by samples at impact is studied using a device designed and built specifically for this purpose. The experimental results have proven that the standard square CAD model structure has allowed for a 45% wt. reduction in material consumption, respectively, and the tubular one has achieved a reduction in material consumption up to 60% wt. compared with the 100% full plain printed (solid) sample reference. Our studies also aimed at extending the international database regarding the optimized printing of model architectures for ABS and polyhydroxyalkanoate composite materials. 

## 2. Materials and Methods 

### 2.1. Materials

PLA and ABS filaments designed for fused filament fabrication have been bought from Verbatim (Eschborn, Germany). A PLA/PHA/Bamboo Fill filament including a composite mixture of PLA, polyhydroxyalkanoate (PHA) and bamboo (Bambusa sp.) wood flour (PLA/PHA/Bamboo Fill) at 20 %wt. loading was bought from ColorFabb (Belfeld, The Netherlands). The diameter of each type of filament was 2.85 ± 0.05 mm. 

These materials were chosen due to their broad availability, excellent extrusion properties, good post-printing modelling ability and tunable texture. 

The printing temperatures for each type of filament (measured at the extrusion head) were 230 °C for the PLA and PLA/PHA/Bamboo Fill filaments and 275 °C for ABS. The bed temperatures for PLA and PLA/PHA/Bamboo Fill filaments were 60 °C and 90 °C for ABS, respectively, as recommended by the manufacturer.

### 2.2. Samples Preparation

**Figure 1:**
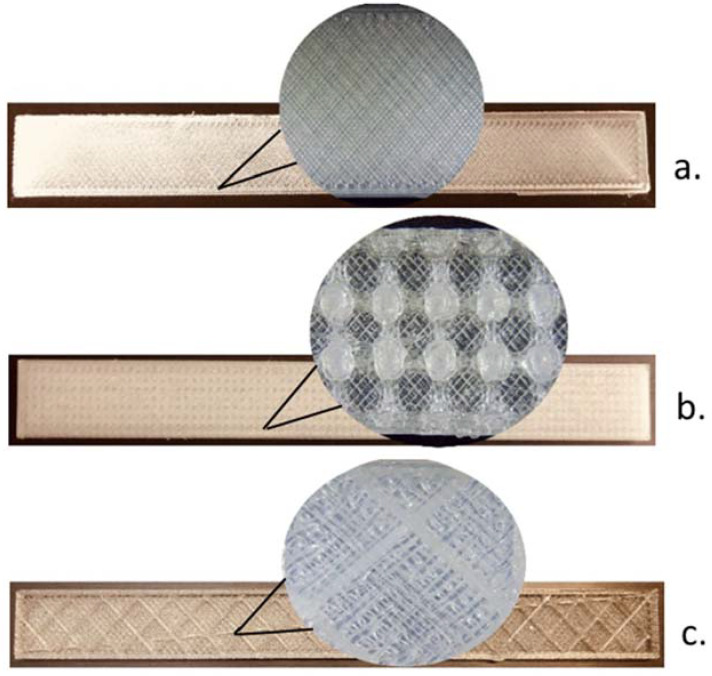
The internal architecture used for the 3D printing of the samples: (**a**). solid, (**b**). cylinder (tube), (**c**). standard.

Different 3D-printing pattern architectures were tested to study their influence on the cross-sectional morphology and mechanical properties of the samples, according to Figure 1.

The specifics of the samples were:

✓ For a: solid (plainly printed), without holes;
✓ For b: Holes were provided on the x, y and z-axis with a diameter of 1.6 mm and 2 mm distance between the axes.
  Plain walls with 0.5 mm thickness were printed to encase these samples;
✓ For c: square holes were provided on the z-axis (4 × 4 mm) and a distance between axes of 4.5 mm.
  Plain walls with 0.5 mm thickness were printed to encase these samples.

From each variant presented in Figure 1, five samples were obtained using a CreatBot DX - 3D double-nozzle printer, using 0.4 mm printing resolution and a 0.2 mm layer resolution for performing each mechanical test. The type and dimensions of the samples were adapted to the tests as follows:

- Tensile test samples: total length 160 mm, calibrated length 60 mm, thickness 3 mm, radius between the calibrated section and the fastening sections 76.29 mm, fastening section length 22.5 mm and 20 mm width;
- Cylindrical blocks for compression tests: diameter 10 mm, height 15 mm;
- Three point-bending samples: 160 mm length, 16 mm width and 3 mm height;
- Impact energy (shock resistance) cubic samples: 15 mm in edge.

In order to eliminate uncertainly values, each mechanical test was replicated five times, and the average values for the mechanical strength of the prints are presented in the paper.

### 2.3. Characterization Methods

There are many applications of materials with high tensile strength and flexibility in consumer FFF-distributed manufacturing. First, many practical applications demand high-strength materials, such as the appropriate open-source technologies (OSAT) for the developing world.

The application of the flexible materials tested here includes shock-absorbing outer coverings on sensitive equipment and improved grip. These materials can also be used to make custom insoles and orthotics. The technical applications of such flexible materials would include timing belts, gaskets, air bladders, O-rings, shock absorption, and vibration dampening.

The tests for tensile, compression, and three-point bending in our research were performed on a universal test machine named WDW- 150 S (Jinan Testing Equipment IE Corporation, Jinan, China). The test machine can perform static and dynamic testing (including alternating fatigue). The applied test force varied between 0.1 and 150 kN. Mechanical tests were performed according to the following standards: ASTM D638, ASTM D790-03 and ASTM E2954-15.

**Figure 2:**
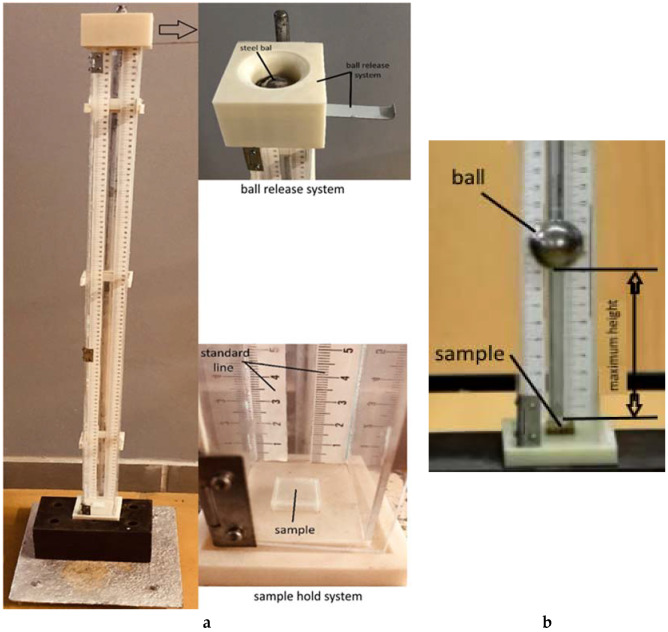
Shock impact device: (**a**). impact testing experimental set-up, (**b**). image analysis and measurement of the maximum height for sample.

For shock impact testing, the device presented in Figure 2 was designed and built in our research center. The device consists of a support holding for a steel ball at a specified height, measured with a ruler. When the ball is released from its support, it falls from a height of 1 m and impacts the sample. To immortalize the impact moment, a high-resolution camera is used to measure the recoil height and the recoil energy of the sample. Details of the impact shock energy determination and calculation could be found in the specific paper [27].

Characteristics of the device: steel ball with m = 99.81 g; rigid steel support (to avoid vibratory motion) with m_1_ = 5 kg.; standard line; high resolution camera; device to release the steel ball.

For the structure and morphology analysis, representative samples have been cross-sectioned, embedded into acrylic resin and levelled using an automatic metallographic Phoenix Beta polishing device from Buehler (with Al_2_O_3_ suspension and 0.05 μm grit).

The optical micrographs were acquired with a Nikon OMNIMET-BUEHLER microscope (Tokyo, Japan) at various magnifications (25× and 100×), specified on each micrograph.

## 3. Results

### 3.1. Mechanical Properties of the Printed Materials

**Figure 3:**
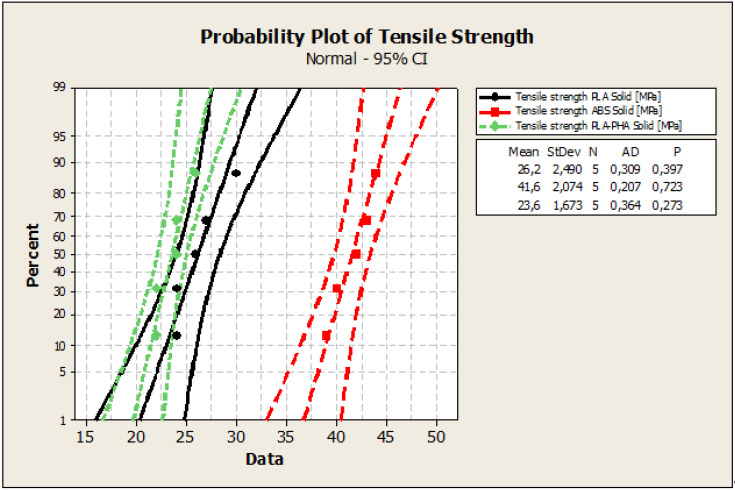
The probability plot of the tensile strength of solid specimens.

The Anderson–Darling statistic test was used to decide if there is a significant relationship between the internal build geometry and the mechanical properties (tensile, compressive, three-point bending strength, impact resistance) of the prints. To verify if a normal distribution describes the mechanical strength data, probability plots were created using Minitab 16 software. In all cases, the appurtenance of the experimental data to the normal distribution was observed. Regarding the tensile tests, an analysis of Figure 3, reveals that the experimental data were close to the straight line and were included in the 95% confidence interval. These results indicated that the data were normally distributed. The P-value of the Anderson–Darling normality test (Figure 3) was: 0.397 (tensile strength - specimens from PLA Solid); 0.723 (tensile strength - specimens from ABS Solid); 0.273 (tensile strength - specimens from PLA-PHA Solid). All data series (the values of tensile strengths for solid, standard and cylindrical tube specimens) passed the Anderson–Darling normality test because they both followed the straight line, and the P values for the normality test were greater than 0.05.

In Table 1, the main statistical indicators which have been described for the specimen with the highest tensile strength of each type of configuration (solid standard and cylindrical elements) are shown. 

In engineering, the variation coefficient of the Anderson–Darling statistic model determines the threshold of transition from a homogenous dataset (between 0%–30%) to heterogeneity (> 30%). The variation coefficients for the prints were between 1%, and 15%, which indicated a good homogeneity of the experimental data. 

**Figure 4:**
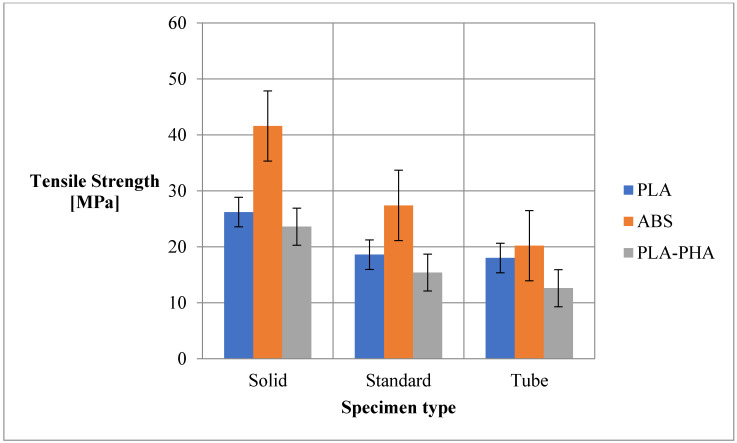
Tensile strength of the 3D printing specimens.

As can be seen from Table 1 and Figure 4, the highest tensile strength was obtained for ABS prints in all configurations (solid, standard and cylindrical tube). Solid samples from ABS present a double high tensile strength compared to solid samples from PLA and PLA/PHA/BambooFill material.

Compared to the data obtained by other researchers [28], the values of tensile strength for ABS obtained by us are higher in the solid configuration but comparable to those obtained by us in the standard configuration with the advantage of reducing material consumption by 45%. In the case of PLA and PLA/PHA BambooFill, the results obtained are close to or slightly lower than those obtained by other authors [28,29,30]

**Figure 5:**
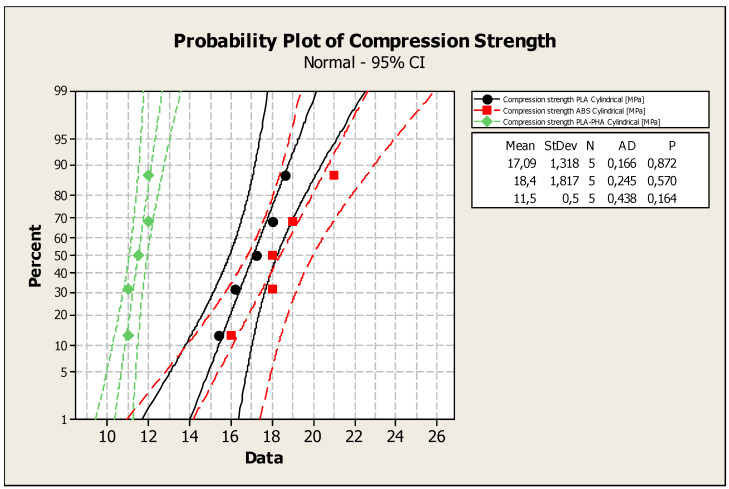
The probability plot for compression strength of the solid specimens

The P-values (Figure 5) of the Anderson–Darling normality test were 0.872 (compression strength - specimens from PLA - cylindrical tube); 0.57 (compression strength - specimens from ABS cylindrical tube), 0.164 (compression strength - specimens from PLA-PHA cylindrical tube). All the data series (the values of compression strengths for solid, standard and cylindrical tube specimens) passed the Anderson–Darling normality test due to their linear dependency, and the P values for the normality test were higher than 0.05.

The statistical indicators for the higher compressive strength values of the specimens with different types of 3D-printed (solid standard and cylindrical) elements are shown in Table 2. The calculated coefficient of variation for all the data from the compression tests is under 30%, so it can be concluded that the experimental data of the tests are homogeneous. At the same time, that is the average of a statistically representative experimental lot. As can be seen from Table 2, the highest tensile strength is recorded for the ABS specimen.

**Figure 6:**
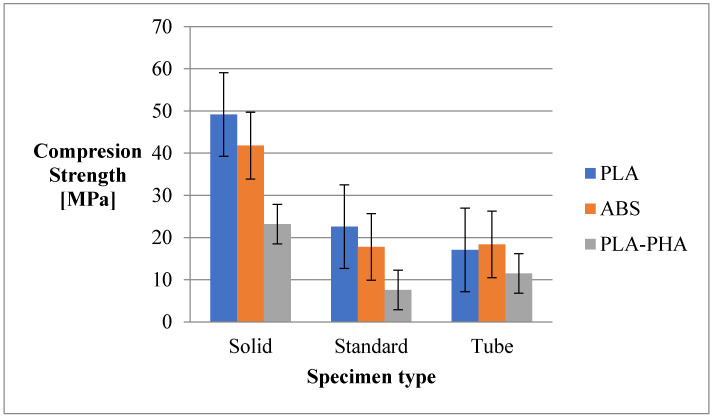
The compression strength of the 3D printing specimens.

As can be seen from Figure 6, the highest compressive strengths are recorded in the case of PLA material, followed by ABS, and lastly by PLA/PHA BambooFill print. The values recorded by the standard configuration and tubes are found at a half of the values recorded at the solid configuration.

The solid configuration is the best choice for parts that are subjected to compression strength.

In the case of the compression strength in general, the results obtained are in agreement with the specialized literature [31] and the properties vary proportionally with the consumption of material. The results in the case of the cylinder tube configuration (for ABS and PLA/PHA) are atypical, where there is a higher compressive strength for the standard configuration and a 15% reduction in material consumption (comparing the standard configuration with the cylinder one).

**Figure 7:**
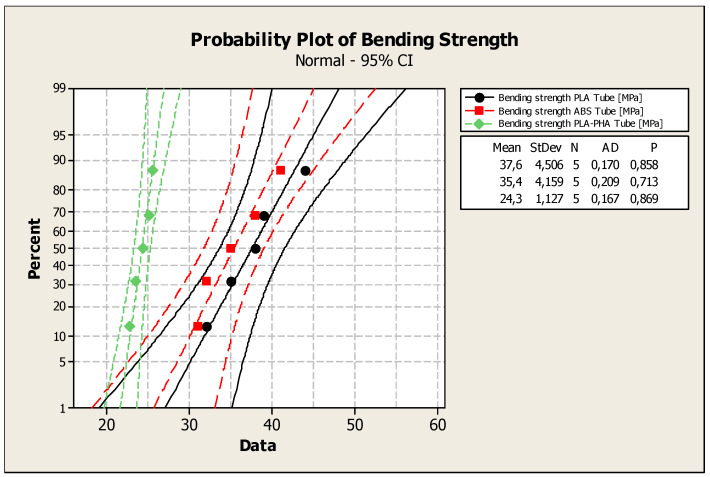
Bending strength probability plot for the cylindrical tube specimens

The *p*-values (Figure 7) of the Anderson–Darling normality test were: 0.858 (bending strength: PLA cylindrical tube); 0.713 (bending strength: cylindrical tube from ABS); 0869 (bending strength: PLA-PHA cylindrical tube).

The statistical indicators for the bending strengths in all of the print variants (solid, cylindrical tube and standard) are shown in Table 3. By analyzing the results of Table 3, it can be concluded that the highest tensile strength is recorded for PLA with all three internal architecture configurations (solid standard and cylindrical elements). The coefficient of variation in the bending strength is lower than 30%, so it can be concluded that the experimental data of tests are homogeneous.

**Figure 8:**
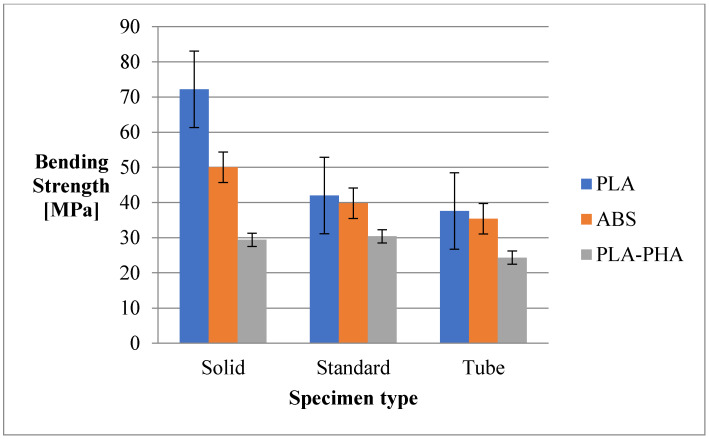
Bending strength of the 3D printing specimens.

As shown in Figure 8, the highest bending strength was recorded for PLA and ABS in the solid configuration.

In all three configurations (solid, standard and tube) the PLA/PHA/BambooFill material registered the lowest values, probably due to the presence of the wood particles in the composition of the print.

Solid samples from PLA are twice as resistant to bending than solid samples from PLA/PHA/BambooFill.

The three-points bending strength is directly influenced by the material consumption (for standard and cylinder configuration) and by the sawdust in the case of PLA/PHA/BambooFill. The sawdust in the PLA/PHA mixture makes the whole composite “fragile” through an uneven distribution in the filament and the greater number of defects and voids, as well as by the non-adhesion between the bamboo particles and the base material at printing.

Regarding to the impact energy, from Figure 8, it can be observed that the datapoints (values of impact energy absorption) can be modelled under the normal distribution assumption. 

**Figure 9:**
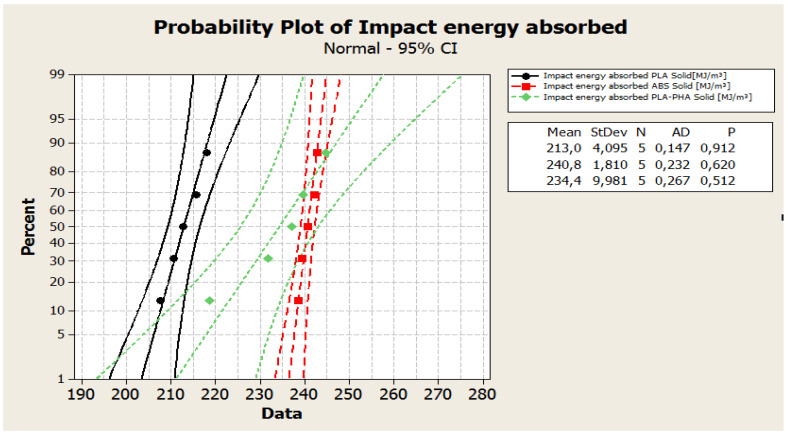
The probability plot for the absorbed impact energy of solid specimens.

The *p*-value (Figure 9) of the Anderson-Darling normality tests for the absorbed impact energy were: 0.912 (PLA in solid configuration); 0.62 (ABS in solid configuration), respectively 0.512 (PLA/PHA/BambooFill in solid configuration). All data series (the values of impact energy absorbed for solid, standard and cylindrical tube specimens) passed the Anderson–Darling normality test and the P values for the normality test were higher than 0.05.

As can be seen from Table 4, high values for impact energy absorption were registered in the 3D-printed PLA–PHA material in both cylindrical tube and standard configurations, as well as for solid PLA. The impact energy increases as the porosity and the number of voids from the internal architecture increase. The coefficient of variation for all the data from the impact energy tests is under 30%, so it can be concluded that the experimental data obtained at impact tests are homogeneous.

**Figure 10:**
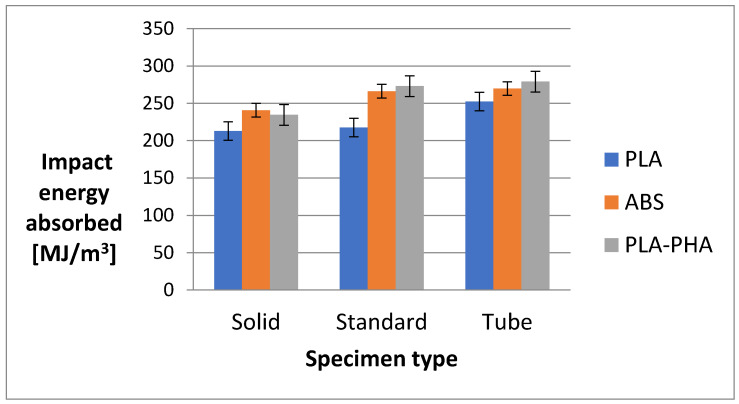
The absorbed impact energy of the 3D printing specimens.

From Figure 10, it can be concluded that solid samples from the PLA-PHA material present the highest impact energy for two types of print architectures (standard and tube).

By using the standard and cylinder internal type configuration, it is possible to obtain higher impact resistance than in the case of the solid configuration, concomitant with a reduction in material consumption of 40% and 60%, respectively. Another factor that has led to the improvement in these properties is the addition of bamboo sawdust, which seems to have a higher energy absorption capacity than other materials (PLA and ABS).

Gaps and/or agglomerations in the material also contribute to a greater absorption of the impact energy.

### 3.2. Metallographic Analysis of the Printed Materials

**Figure 11:**
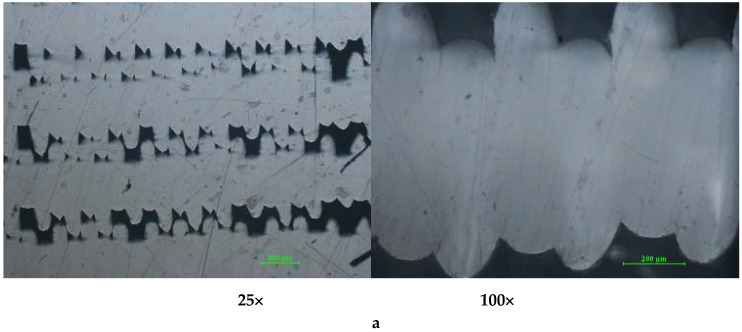

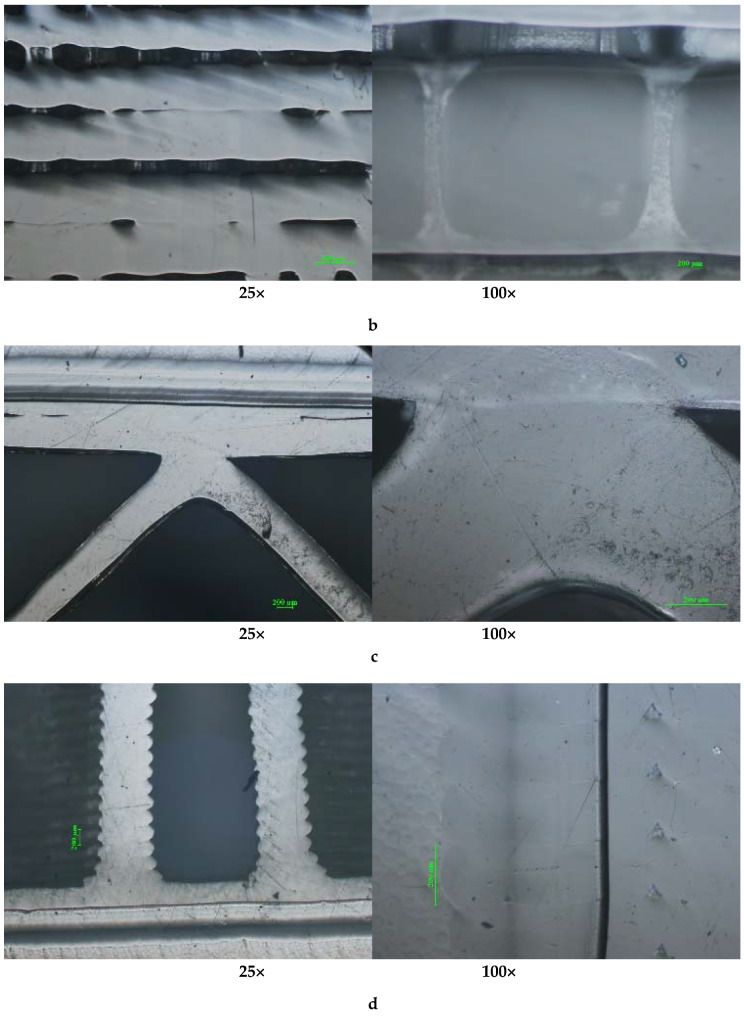

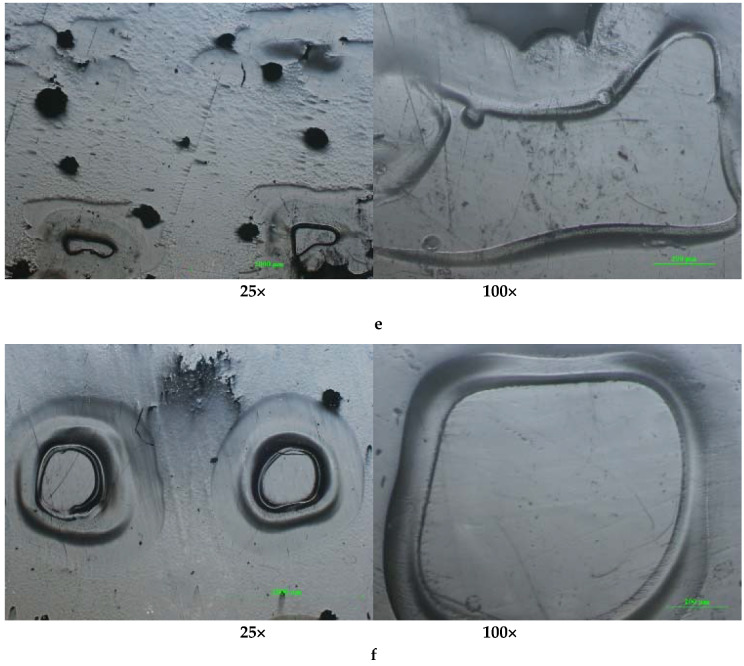
Cross-section optical micrographs of PLA samples at 25× and 100× magnification. (**a**) Solid vertical wall without holes; (**b**) Solid horizontal wall without holes. (**c**) Cylindrical tube vertical wall; (**d**) Cylindrical tube horizontal wall (**e**) Standard vertical wall; (**f**) Standard horizontal wall.

As can be seen in the micrographs from Figure 11a–f, the PLA prints show a compact mass without any macrostructural defects. The cross-sections of the printed samples present several minor defects (inter-layer pores), with regular triangular patterns in the case of PLA (Figure 11a,b,f).

The fewest defects occur in the case of the PLA-printed sample, where the deposited filament can merge better into coalesced layers due to the low value of the PLA melt/air interfacial tension, resulting in a reduction. The combination of the solidification rate of the melted polymer and the elimination of gases entrapped air results in smaller, closed pores of more regular shape. [27].

**Figure 12:**
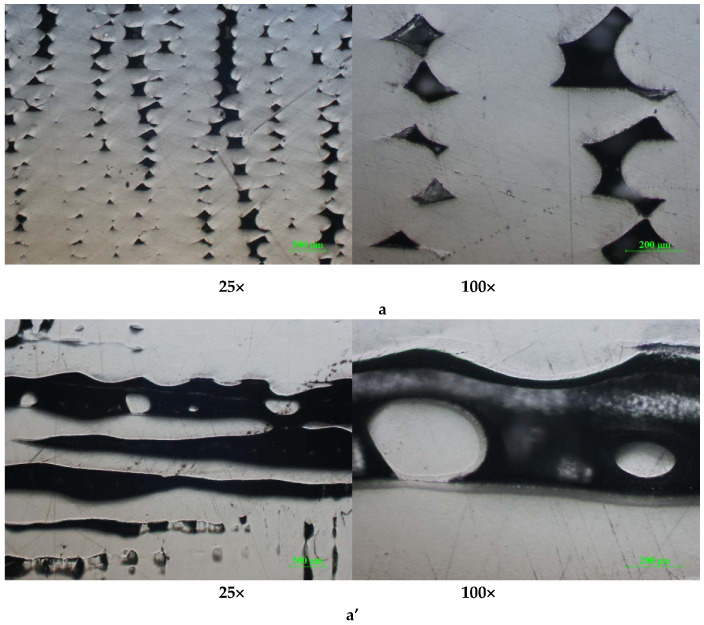
Cross-section optical micrographs of ABS samples at 25× and 100× magnification. (**a**). Solid vertical wall without holes; (**a’**). Solid horizontal wall without holes.

**Figure 13:**
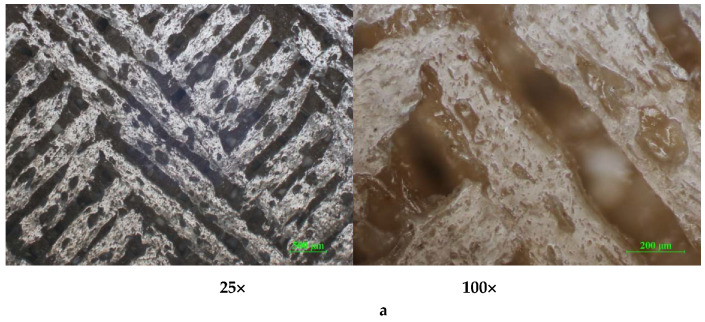

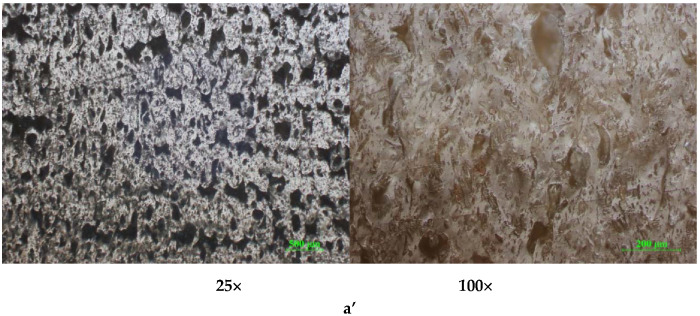
Cross-section optical micrographs of PLA/PHA BambooFill samples at 25× and 100× magnification. (**a**). Solid vertical wall without holes; (**a’**). Solid horizontal wall without holes.

The cross-sections of the printed samples present several defects (interlayer pores), with irregular patterns in the case of ABS (Figure 12a,a’)) and PLA/PHA/BambooFill (Figure 13a,a’)), caused by the inclusion of small air bubbles and agglomeration of sawdust powder (uneven distribution).

At higher temperatures (for ABS), the previously deposited layers remain in a softened state, which leads to a nonhomogenity in the thickness of the layers as well as a reduction in the dimensional precision.

In contrast with PLA, in the PLA/PHA/BambooFill, the density of defects is the highest, due to the localized influence of the wood particles on the polymer layer distribution and adhesion. 

Another factor that causes defects is the temperature of the build platform (printing bed). At a lower temperature (such as for PLA and PLA/PHA/BambooFill), the bonding between layers is faulty due to the solidification of the previously deposited layers, resulting in voids, pores, and poor adhesion between layers (exfoliation).

## 4. Conclusions

In this paper, the mechanical properties of PLA, ABS and PLA / PHA BambooFill were obtained with three internal configurations (solid, standard and cylinder (tube)).

The statistical analysis of the tensile, compression, 3-point bending and impact resistance have permitted the highlighting of the following recommendations regarding the material type: PLA, ABS or PLA/PHA/BambooFill, and the architecture of the model to be printed.

✓ For parts subjected to tensile strain: it is recommended to use ABS, PLA in solid configuration, followed by ABS in standard configuration when high strengths are required. Using the standard configuration and the ABS material, tensile strengths can be obtained, with approximately 20% higher from the point of view of the material consumption ratio / tensile strength;
✓ For parts subjected to compression: it is recommended to use PLA, ABS and PLA/PHA/BambooFill materials in solid configuration. The cylindrical architecture confers a higher compressive strength compared to the standard architecture and a 15% reduction in material (comparing the cylindrical with standard consumption);
✓ For parts subjected to flexural stresses: it is recommended to use PLA, ABS materials in solid configuration, followed by PLA in standard configuration. The use of PLA / PHA BambooFill material is not recommended due to its low properties in parts with this type of request;
✓ For excellent impact resistance: it is recommended to use PLA/PHA/BambooFill material in tube configuration, followed by ABS in standard configuration. The internal architecture of the cylindrical type presents the best properties for energy absorption and gives a 65% reduction in material consumption;
✓ The mechanical behavior of FFF parts is primarily determined by the material type and, only after that, the internal architecture.

The longitudinal tension directed toward the cylinder axis is simply calculated, on the basis of the basic theory of tensile testing, taking into account the fact that the internal pressure produces a stretching force and the outer pressure is compressive. This strength is constant and evenly distributed over the tube thickness.

The internal architecture of the tube also gives it a high radial resistance. It is known that the radial–axial resistance of a tube is much higher compared to the full tube, which is actually a bar, despite the fact that its weight with internal architecture is much smaller than the filled bar.

Furthermore, the inner resistance can be calculated as a tube with the inner lattice, these being represented by the dimensions of the architectural forms.

A reduction in material consumption, while preserving as much as possible the mechanical strength, is a long-standing desiderate of additive manufacturing. In this direction, choosing the standard configuration leads to a reduction of 45% in filament consumption, while for the tube configuration, this reduction can reach 60%.

Further research is needed in order to create new internal architectures, with a more significant reduction in the weight of the pieces and perhaps with much better mechanical characteristics.

The results presented in the paper could give a useful insight into the behavior of those materials used for additive manufacturing with different internal architectures and could enlarge the existent international database related to the processing–structure–mechanical properties of these materials.

The study clearly demonstrates that the tensile strength of a 3D-printed specimen depends largely on the mass of the specimen, for all materials. This dependence enables prosumers to solve the challenge of unknown print quality effects on the mechanical properties of a 3D-printed part using a two-step process to estimate the tensile strengths for a given material. 

First, the exterior of the print is inspected visually for sub-optimal layers. Then, to determine if there has been under-internal architecture, the mass of samples is measured. This mass is compared to what the theoretical value is using the densities provided in this study for the material and the volume of the sample. This two-step process provides a means to assist low-cost open-source 3D printers and expand their range of sample production to functional parts. 

## Figures and Tables

**Table 1:** Statistical indicators for the tensile tests of the specimens.

Specimen Type	Mean [MPa]	Variance [MPa^2^]	Standard Deviation [MPa]	Coefficient of Variation [%]
ABS—Solid	41.6	4.3	2.074	4.98
ABS—Standard	27.4	0.3	0.548	2
ABS—Cylindrical Tube	20	4.2	2.049	10.15

**Table 2:** Statistical indicators for the compression tests of the specimens.

Specimen Type	Mean [MPa]	Variance [MPa^2^]	Standard Deviation [MPa]	Coefficient of Variation [%]
PLA—Solid	49.2	23.71	4.87	9.9
PLA—Standard	22.576	0.53	0.728	3.23
ABS—Cylindrical Tube	18.4	3.3	1.817	9.87

**Table 3:** Statistical indicators for the bending tests of the specimens.

Specimen Type	Mean [MPa]	Variance [MPa^2^]	Standard Deviation [MPa]	Coefficient of Variation [%]
PLA—Solid	72.2	3.2	1.789	2.48
PLA—Standard	42	4	3	4.76
PLA—Cylindrical Tube	37.6	20.3	4.51	11.98

**Table 4:** Statistical indicators determined through the impact energy tests of the specimens.

Specimen Type	Mean [MJ/m^3^]	Variance [MJ/m^3^]	Standard Deviation [MJ/m^3^]	Coefficient of Variation [%]
PLA—Solid	240.78	3.27	1.81	0.75
PLA-PHA—Standard	272.93	31,25	5.59	2.05
PLA-PHA—Cylindrical Tube	279.01	56.42	7.51	2.69
